# Clinical-histopathologic features in oral lichen planus and lichenoid lesions: A comparative analysis

**DOI:** 10.6026/973206300220192

**Published:** 2026-01-31

**Authors:** Navneet Singh Kathuria, Gagandeep Kaur Sidhu, Navdeep Singh Kathuria, Amandeep Kaur, Rahul Sharma

**Affiliations:** 1 Department of Oral Pathology, Dr S.S. Tantia Dental College, Hospital and Research Centre, Sriganganagar, Rajasthan, India; 2 Department of General Medicine, Dr S.S. Tantia Dental College, Hospital and Research Centre, Sriganganagar, Rajasthan, India; 3 Department of Conservative and Endodontics, Dr S.S. Tantia Dental College, Hospital and Research Centre, Sriganganagar, Rajasthan, India; 4 Department of Oral and Maxillofacial Surgery, Dr S.S. Tantia Dental College, Hospital and Research Centre, Sriganganagar, Rajasthan, India

**Keywords:** Oral lichen planus, oral lichenoid lesions, histopathology, diagnosis, eosinophils, oral pathology

## Abstract

The clinical and histopathological distinctions between oral lichen planus (OLP) and oral lichenoid lesions (OLLs) are clinically and histopathologically difficult since they both have overlapping clinical presentations. The retrospective cross-sectional study involved the assessment of 100 biopsy-proven cases to determine discriminating clinical patterns and microscopic characteristics. OLP were significantly more bilateral and had a typical band-like superficial lymphocytic infiltrate with OLLs having recognisable triggers, more inflammation around vessels and frequent eosinophils. These histopathologic clues had high levels of discriminatory power between the two entities. A combination of clinical and major microscopic markers, i.e., eosinophil-rich infiltrates has a great impact on diagnostic accuracy between OLP and OLL.

## Background:

Oral lichen planus (OLP) is an oral mucosa condition that is rather frequent, chronic, inflammatory, and mucocutaneous with no skin manifestations in many cases [1]. It is believed to be an unknown etiology T-cell-mediated autoimmune disease that has a number of clinical phenotypes including reticular, papular, plaque-like, atrophic, erosive, and bullous type [2]. OLP diagnosis is highly relevant to the management of the patient as it is symptomatic, has a chronic path, remissions and exacerbations, and is classified by the World Health Organization (WHO) as an oral potentially malignant disorder [3]. To make the matters worse, there are oral lichenoid lesions (OLLs), which is a range of conditions that cannot be clinically or histopathologically distinguished or are very similar to OLP [4]. The OLLs, however, do not occur idiopathically; they are often related to a particular etiologic agent. They are hypersensitivity responses to dental materials (e.g., amalgam, gold, composite resins) which are called oral lichenoid contact reactions (OLCRs), and reactions to a number of systemic drugs, called oral lichenoid drug reactions (OLDRs) [5, 6]. The list of involved drugs is long and includes antihypertensives, non-steroidal anti-inflammatory drugs (NSAIDs), hypoglycemic agents among others. The difference between OLP and OLL is a clinical issue of the highest relevance. When an OLL is accurately diagnosed, the causative agent can be eliminated, in case of a dental restoration, or by switching a drug, which will cause the lesion to heal [7]. Conventionally, OLP management includes the long-term symptomatic treatment, normally using topical or systemic corticosteroids and necessitates the regular long-term checking of malignant transformation, which is reported as 1-2 per cent in occurrence [8].

Although this is a dire need of differentiation, there is a lot of controversy and uncertainty over clear-cut diagnostic criteria. Clinical manifestation of OLLs may be the same as that of OLP, but in some cases, OLLs are said to be more common and in direct topographic association with the perceived trigger [9]. Both conditions have histopathologic characteristics of characteristic densely banded, below-epithelial lymphocytic subepithelial infiltrate, basal cell liquid degeneration, as well as the presence of the Civatte bodies [10]. To resolve this ambiguity, van der Meij and van der Waal suggested modified diagnostic criteria based on which cases are classified as typical OLP and lesions compatible with OLP but the gray zone is still large [11]. In some studies, it has been argued that some minor histopathologic characteristics including a deeper penetration of inflammatory infiltrate or the presence of eosinophils and plasma cells can be more typical of OLLs [12]. Therefore, it is of interest to conduct a thorough comparative analysis of clinical and histopathologic appearance of a clearly defined group of patients with OLP and OLL in a retrospective manner to infer and measure the most dependable characteristics of differentiating between these two closely related units.

## Materials and Methods:

## Study design and sample selection:

A total of 100 cases with adequate clinical information and high-quality biopsy specimens were selected for inclusion and divided into two groups:

[1] OLP Group (n=50): Patients with a clinical presentation of bilateral, symmetric lesions and no identifiable local or systemic trigger.

[2] OLL Group (n=50): Patients with lesions in close topographic relationship to a dental restoration and/or a clear history of starting a new systemic medication temporally related to the lesion onset.

## Inclusion criteria:

[1] A definitive clinical diagnosis leading to group allocation.

[2] An incisional biopsy report from the lesion.

[3] Availability of original hematoxylin and eosin (H & E)-stained slides and paraffin-embedded blocks.

[4] Complete clinical records with demographic data, lesion description, and relevant medical/dental history.

## Exclusion criteria:

[1] Cases of graft-versus-host disease.

[2] Patients with a concurrent diagnosis of another vesiculobullous disease (e.g., pemphigoid).

[3] Biopsy specimens with significant crush artifact or insufficient epithelial tissue for evaluation.

[4] Incomplete or ambiguous clinical records.

## Data collection:

[1] Clinical data: The following information was extracted from patient charts: age, gender, lesion site(s), clinical morphology (reticular, erosive, etc.), lesion symmetry (unilateral vs. bilateral), and the presence of a suspected trigger (amalgam restoration, new medication).

[2] Histopathologic evaluation: All H & E-stained slides were re-evaluated by two board-certified oral pathologists who were blinded to the original diagnosis and clinical group allocation. Any disagreements were resolved by consensus discussion at a dual-headed microscope.

[3] The following features were recorded as present or absent:

1) Epithelial changes: Hyperkeratosis (ortho- or para-), epithelial atrophy or hyperplasia, acanthosis, and presence of "saw-tooth" rete pegs.

2) Interface changes: Liquefactive degeneration of the basal cell layer and presence of apoptotic keratinocytes (Civatte bodies).

3) Inflammatory infiltrate:

a) Density: Mild, moderate, or dense.

b) Distribution: A continuous, dense, band-like infiltrate confined to the superficial lamina propria.

c) Depth: Presence of a deep, perivascular inflammatory infiltrate extending into the deeper connective tissue.

d) Composition: Predominantly lymphocytic, or mixed with a notable presence (defined as >10 cells per high-power field) of plasma cells and/or eosinophils.

## Statistical analysis:

Data were entered and analyzed using SPSS version 27.0 (IBM Corp.). Descriptive statistics were generated for all variables. The Chi- square test or Fisher's exact test was used to compare the frequencies of categorical clinical and histopathologic features between the OLP and OLL groups. The independent samples t-test was used to compare the mean age between the two groups. A p-value of less than 0.05 was considered statistically significant.

## Results:

The study included 100 patients, with 50 in the OLP group and 50 in the OLL group. The mean age of patients in the OLP group (56.8 ± 12.1 years) was slightly higher than in the OLL group (52.5 ± 14.5 years), but this difference was not statistically significant (p=0.11). Both groups had a female predilection (68% in OLP, 62% in OLL). As shown in Table 1, there were significant differences in clinical presentation. Bilateral and/or symmetric lesions were overwhelmingly more common in the OLP group (88.0%) compared to the OLL group (32.0%) (p<0.001). Conversely, a clear identifiable trigger was noted in 46.0% of OLL cases (25% related to amalgam, 21% to medication) but in only 4.0% of OLP cases (p<0.001). The buccal mucosa was the most common site in both groups. The frequencies of various histopathologic findings are detailed in Table 2. Several classic features were highly prevalent in both groups but more consistently seen in OLP. A dense, band-like lymphocytic infiltrate was observed in 96.0% of OLP cases versus 70.0% of OLL cases (p=0.001). Liquefactive degeneration of the basal cell layer was also very common in both (92.0% in OLP, 80.0% in OLL), with no significant difference (p=0.08). The most significant distinguishing features related to the depth and composition of the inflammatory infiltrate. A deep, perivascular inflammatory component was found in 64.0% of OLLs, compared to only 10.0% of OLP cases (p<0.001). The presence of eosinophils was a powerful discriminator, identified in 58.0% of OLL biopsies but only 6.0% of OLP biopsies (p<0.001). A significant plasma cell infiltrate was also more common in OLLs (38.0% vs. 12.0%, p=0.003). The distribution of clinical subtypes is shown in Table 3. Reticular lesions were the most common presentation in both groups. Erosive lesions were slightly more frequent in the OLL group, but this difference did not reach statistical significance (p=0.22). This highlights the substantial clinical overlap between the two conditions.

## Discussion:

The OLP-OLL distinction is a longstanding diagnostic dilemma of oral patients and pathology. This paper presents a quantitative data on the use of a combined clinical approach and histopathologic approach in order to differentiate these entities. Our results support the fact that although there is a high level of overlap, some features are highly and significantly related to one of the conditions rather than the other. Our findings are in agreement with the past reports that classic OLP is usually bilateral and symmetric lesions, and OLLs are more commonly unilateral [13]. The most important clinical data is the positive correlation OLLs have with recognizable trigger which occurred in close to half of our OLL cohort. An elaborate patient history on new drugs or installation of dental restorations is thus unavoidable and should be the initial procedure in diagnosis process [7]. Bilateral lesions combined with the lack of a trigger should increase the index of suspicion of idiopathic OLP. Our very study center is in the histopathologic analysis, however. Although both groups shared the classic features characterized by the modified WHO criteria; band-like lymphocytic infiltrate and basal cell liquefaction, the features were more consistent among the OLP [11]. It implies that they are required to be present to make a "lichenoid" diagnosis, but their absence in a clinically suspicious lesion may indicate an exclusion of classic OLP. The strongest histopathologic features that we found in our study were not usually highlighted in the conventional definition of OLP: the depth of inflammation and the presence of eosinophils. It is of vital importance that in 64 percent of the OLLs and only in 10 percent of the OLP cases there was a deep, perivascular infiltrate. This implies a variant of the inflammatory process, which is not necessarily limited to the epithelial-connective tissue interface [14].

Better still, there were eosinophils. One of the findings is their identification in 58 percent of the OLL cases as opposed to 6 percent of the OLP cases. Eosinophils are normally related to Type I and IV hypersensitivity reactions [12]. Their occurrence in OLLs follows the prediction of mechanopathy between them and OLP and is a form of hypersensitivity reaction to some external antigen, including a drug hapten or a metallic ion in a dental amalgam [10]. A major eosinophilic mediator is not generally a part of the autoimmune pathogenesis of OLP, which is T-cell mediated [15]. Therefore, the presence of over a few scattered eosinophils within a lichenoid infiltrate must give way to a deep investigation of the existence of an exogenous cause. Our findings are consistent with and support quantitatively the measures used by other researchers such as Ho and his associates who indicated that the presence of eosinophils, plasma cells and deep infiltrate are useful indicators of the diagnosis of OLLs [9]. The role of this difference cannot be overestimated. It has a direct effect on management as it leads the clinician to trigger elimination in OLL, and notifies the necessity of long-term malignancy surveillance in OLP [8]. There are limitations of this study. It is retrospective, and this will depend on the quality of the current records. The first clustering was done on clinical criteria, which is vulnerable to a certain degree of circularity, but the re-evaluation of the blind histopathologists was meant to reduce this bias. Additionally, this was a single-centered study, and the practice patterns could be different. More understanding of the immunopathogenic distinctions between OLP and OLL might be obtained by future prospective studies that employ more advanced methods such as direct immunofluorescence analysis of immunoglobulin and complement deposition, or immunohistochemistry analysis of the lymphocyte subtypes [16, 17].

## Conclusion:

We show that it is only through a critical synthesis of clinical and histopathologic evidence that a reliable diagnosis of oral lichen planus versus oral lichenoid lesions could be made. Whereas OLP is either bilateral or idiopathic, OLLs are unilateral and accompanied by a trigger. Histopathologically, a deep, perivascular, inflammatory infiltrate and, most importantly, the presence of eosinophils are powerful and dependable signs of an OLL.

## Figures and Tables

**Table 1:** Comparison of clinical and demographic features

Feature	OLP Group (n=50)	OLL Group (n=50)	p-value
Mean Age (years) ± SD	56.8 ± 12.1	52.5 ± 14.5	0.11
Female Gender, n (%)	34 (68.0%)	31 (62.0%)	0.53
Bilateral/Symmetric Lesions, n (%)	44 (88.0%)	16 (32.0%)	<0.001
Identifiable Trigger, n (%)	2 (4.0%)	23 (46.0%)	<0.001
Buccal Mucosa Involvement, n (%)	47 (94.0%)	43 (86.0%)	0.15

**Table 2:** Comparison of key histopathologic features

Histopathologic Feature	OLP Group (n=50)	OLL Group (n=50)	p-value
Dense, Band-like Infiltrate	48 (96.0%)	35 (70.0%)	0.001
Basal Cell Liquefaction	46 (92.0%)	40 (80.0%)	0.08
Civatte Bodies	39 (78.0%)	31 (62.0%)	0.09
"Saw-tooth" Rete Pegs	28 (56.0%)	21 (42.0%)	0.16
Deep, Perivascular Infiltrate	5 (10.0%)	32 (64.0%)	<0.001
Eosinophils Present	3 (6.0%)	29 (58.0%)	<0.001
Plasma Cells Present	6 (12.0%)	19 (38.0%)	0.003

**Table 3:** Distribution of clinical morphologies

Clinical Type	OLP Group (n=50)	OLL Group (n=50)	p-value
Reticular/Papular only	29 (58.0%)	23 (46.0%)	0.23
Erosive/Atrophic	18 (36.0%)	23 (46.0%)	0.22
Plaque-like	3 (6.0%)	4 (8.0%)	0.72
